# Computed Tomography Angiography-Derived Pericoronary Fat Attenuation Index and AI-Based Cardiovascular Risk Score as a Proposed New Pathway in a Rapid Access Chest Pain Clinic: A Single-Centre Retrospective Registry Study

**DOI:** 10.3390/jcm15218639

**Published:** 2026-11-06

**Authors:** Nerea Sanfeliu Garces, Ahmed Alsinbili, Lucas Costa, Tracy Barnfather, Lynne Cooley, Barbara Kardos, Mike Pashler, Attila Kardos

**Affiliations:** 1 Department of Cardiology, Translational Cardiovascular Research Group, Milton Keynes University Hospital NHS Foundation Trust, Milton Keynes MK6 5LD, UK; 2 Faculty of Medicine and Health Sciences, University of Buckingham, Buckingham MK18 1EG, UK; 3 Department of Radiology, Milton Keynes University Hospital NHS Foundation Trust, Milton Keynes MK6 5LD, UK

**Keywords:** pericoronary fat attenuation index, coronary inflammation, cardiac CT angiography, chest pain, cardiovascular risk stratification, CariHeart, artificial intelligence, preventive cardiology

## Abstract

**Background:** As part of the National Health Service England new chest pain pathway implementation project, we tested the impact of an artificial intelligence (AI)-based, CariHeart risk score-based new pathway (NP) compared with the current pathway (CP). **Methods:** One hundred and thirty-five consecutive patients referred to the Rapid Access Chest Pain Clinic who underwent Cardiac Computed Tomography Angiography (CCTA) for the assessment of suspected coronary artery disease (CAD) were included. CCTA images were analysed for qualitative plaque characterisation and grading of coronary artery stenosis and CariHeart risk score (derived from the perivascular fat attenuation index (FAI) score, plaque burden and clinical risk factors) as part of this analysis. We assessed the proportion of patients with non-obstructive CAD classified as high-risk by CariHeart and the differences between the pathways in initiation/intensification and de-escalation of cardiovascular preventive therapy (CVPT; statin, aspirin, ezetimibe and colchicine). CP was based on nurse assessment and CCTA with subsequent cardiologist recommendation and the NP was based on the same in addition to the CariHeart risk assessment-based management. **Results:** The average age was 61 years, with 47% female and 82% Caucasian. CCTA showed that 83% of patients had non-obstructive CAD (no or <50% stenosis), 16% had moderate CAD (50–69% stenosis), and 1.5% had severe or chronic total occlusion. The CariHeart score indicated low risk (<1% risk) in 3%, intermediate risk (1–5%) in 38%, and high risk (>5%) in 59% of patients. Based on the CariHeart risk score, 54% of patients with non-obstructive CAD were classified as high-risk, whereas 17% of patients with obstructive (≥50%) CAD were classified as intermediate-risk and none as low-risk. Among 133 patients with recommendations under both pathways, escalation of CVPT was recommended for 30% with the CP and 84% with the NP (McNemar *p* < 0.0001); the rest continued their existing therapy. Under the CP, escalation was more frequent in patients with higher FAI score centiles and higher CariHeart risk; under the NP, escalation rates were high in all categories. No de-escalation of medication was recommended in either of the pathways. In patients with no or mild CAD on the local CCTA report, escalation was recommended more often with the NP than with the CP (86% vs. 25%). **Conclusions:** Incorporating the AI-based CariHeart risk score into the chest pain pathway classified a high proportion of patients with non-obstructive CAD as high-risk and was associated with a higher rate of recommended escalation of CVPT than the CP. Whether this improves clinical outcomes requires prospective evaluation.

## 1. Introduction

Ischaemic heart disease (IHD) remains the leading cause of premature death and disability in the United Kingdom, accounting for approximately 64,000 deaths annually and affecting an estimated 2.3 million people [1,2]. Chest pain constitutes one of the most frequent reasons for both emergency department attendance and urgent outpatient referral, placing a substantial and recurrent demand on secondary care diagnostic services. Accurate and timely risk stratification is therefore paramount, both to identify patients requiring intervention and to avoid unnecessary investigation and associated patient anxiety.

In 2010, and following revision in 2016, the National Institute for Health and Care Excellence (NICE) recommended Cardiac Computed Tomography Angiography (CCTA) as the preferred first-line investigation for the assessment of suspected stable coronary artery disease (CAD) in the United Kingdom [3]. This recommendation was underpinned by the Scottish Computed Tomography of the Heart (SCOT-HEART) trial, which demonstrated that CCTA-guided management reduced the five-year rate of fatal and non-fatal myocardial infarction compared with standard care and established CCTA as a powerful tool for anatomical plaque characterisation [4,5]. CCTA offers high sensitivity and negative predictive value for the exclusion of obstructive CAD and has substantially improved diagnostic accuracy and clinical efficiency within rapid access chest pain clinic pathways, referred to here as the current pathway (CP).

However, a fundamental limitation of anatomical CCTA is its inability to characterise the inflammatory biology of the coronary arterial wall. Atherosclerosis is intrinsically an inflammatory process [6], and as many as 80% of patients presenting with chest pain have non-obstructive coronary anatomy on CCTA. Crucially, over 60% of major adverse cardiac events occur in whom a “normal” CCTA result may paradoxically confer false reassurance [7]. The Oxford Risk Factors and Non-Invasive Imaging (ORFAN) study, which followed 3393 consecutive patients undergoing clinically indicated CCTA for a median of 7.7 years, demonstrated that elevated pericoronary inflammation, as quantified by the fat attenuation index (FAI), was independently associated with incident cardiovascular events, irrespective of stenosis severity on CCTA [7]. These observations underscore the need for a complementary imaging biomarker that captures residual inflammatory risk beyond the anatomical assessment provided by conventional CCTA.

The pericoronary FAI is an established quantitative imaging biomarker derived from the pericoronary adipose tissue (PCAT) surrounding the major epicardial coronary arteries, measurable from standard contrast-enhanced CCTA without additional acquisition protocols or radiation exposure [8]. Inflammatory signalling within the coronary arterial wall promotes the release of pro-inflammatory cytokines into the surrounding PCAT, inducing a lipolytic phenotypic shift that increases adipose tissue CT attenuation, thereby enabling non-invasive quantification of coronary inflammation [8,9]. The prognostic validity of FAI was established in the Cardiovascular RISk Prediction using Computed Tomography (CRISP-CT) study, in which elevated pericoronary FAI was independently associated with cardiac mortality over a median follow-up of 54 and 72 months in two prospective cohorts comprising more than 3900 patients in Europe and the United States [8]. A systematic review and meta-analysis have further confirmed the diagnostic and prognostic utility of FAI as an imaging biomarker across diverse clinical settings [9].

Building upon the established prognostic value of FAI, an artificial intelligence (AI)-based risk scoring platform, CariHeart, by Caristo Diagnostics ltd (Oxford, UK), has been developed and validated to generate an individualised eight-year probability of cardiac death [8,10,11]. Because raw pericoronary attenuation varies with scan settings such as tube voltage, and with age and sex [12], its first output is a standardised FAI score for each coronary artery, in which raw FAI is weighted for technical scan parameters, anatomical factors and basic demographics (age and sex). The FAI score still increases with age, so individual values are interpreted against age, sex and vessel-specific nomograms [11]. The FAI score is then combined with coronary plaque burden and clinical risk factors (diabetes, smoking, hyperlipidaemia and hypertension) to estimate the eight-year risk of cardiac death (CariHeart risk) [7,11]. The CariHeart risk score has received regulatory clearance, and its clinical implementation has been piloted across five National Health Service (NHS) hospital sites as part of a national evaluation of a new chest pain pathway, the new pathway (NP). A single-centre study demonstrated that FAI-based risk stratification identifies a substantially greater proportion of at-risk patients compared with QRISK-3 score [10].

Outside the development cohorts, the FAI score and CariHeart risk have been examined in routine practice. In ORFAN, making the AI-risk classification available to clinical teams changed management in 45% of 744 consecutive patients in four NHS hospitals, mainly through initiation or intensification of statin therapy [7]. In a Dutch single-centre study, perivascular FAI assessment changed the estimated cardiovascular risk category in 62% of 50 patients at the highest estimated risk [13]. In a UK lipid clinic, high FAI scores were found across coronary calcium and stenosis categories, including in patients without overt CAD, and in a lipidologist survey CCTA findings changed lipid targets in a further 19% of cases beyond calcium scoring [14]. FAI scores have also been related to the extent of coronary atherosclerosis [15] and only weakly to coronary calcium, with inflamed plaques reported in patients with low or zero calcium scores [16]. How the CariHeart risk changes preventive therapy when it is added to the NHS chest pain pathway has, however, only been reported to a limited extent.

The present study reports the experience of a single secondary care centre in the United Kingdom, as one of the five sites participating in the national pilot programme to assess and facilitate implementation of a new chest pain pathway nationwide. We sought to (i) describe how often patients with non-obstructive CAD on CCTA were classified as high-risk by the CariHeart risk score used in the NP, compared with anatomical grading in the CP, and (ii) to assess the practice-changing potential of the NP on cardiovascular preventive therapy (CVPT).

## 2. Methods

### 2.1. Study Design and Setting

This was a single-centre retrospective registry study of consecutive patients referred to the Rapid Access Chest Pain Clinic (RACPC) at Milton Keynes University Hospital NHS Foundation Trust, in Milton Keynes, as part of the NHS England new chest pain pathway implementation project. The study period encompassed January to September 2024, capturing all patients assessed in the clinic who underwent contrast-enhanced CCTA during this period. The study was designed and conducted in accordance with the principles of the Declaration of Helsinki. All patients provided informed consent for the use of their anonymised imaging data for research purposes and for AI-assisted analysis. Data were anonymised prior to transfer and are reported in aggregate, compliant with UK General Data Protection Regulation and NHS Information Governance standards.

### 2.2. Study Population

The inclusion criteria comprised all adult patients referred to the RACPC with a presenting complaint of chest pain or suspected angina who underwent contrast-enhanced CCTA as part of the current chest pain pathway. The exclusion criteria were limited to absence of intravenous contrast administration (at patient request) and an estimated Glomerular Filtration Rate (eGFR) <30 mL/min/1.73 m^2^. Of 137 consecutive patients enrolled, two were excluded on this basis, yielding an analytic cohort of 135 patients. Patient demographics, cardiovascular risk factors, presenting symptoms, and baseline medications were recorded from electronic clinical records.

### 2.3. Chest Pain Pathways

The current pathway (CP) is the established RACPC pathway, which is run by advanced nurse practitioners with indirect consultant supervision; after clinical assessment and CCTA, management was recommended by a consultant cardiologist on the basis of the clinical findings and the local CCTA report. In the new pathway (NP), the same assessment was supplemented by the CariHeart analysis, and the CariHeart report was used by a cardiologist in the treatment decision. CP and NP recommendations were recorded for each patient so that each patient contributed to both pathways. Two measures of preventive therapy are reported: the treatments listed in the CariHeart report, and the treatments initiated or intensified under each pathway, classified against the patient’s baseline therapy.

### 2.4. Cardiac Computed Tomography Angiography and FAI Score Analysis

All CCTA examinations were performed according to standard institutional protocols using established acquisition parameters appropriate for coronary imaging. Contrast-enhanced CCTA images were anonymised locally and transmitted securely to Caristo Diagnostics (Oxford, UK) for AI-assisted pericoronary FAI analysis. The CariHeart platform quantified the pericoronary fat attenuation index for each of the three major coronary arteries: the Left Anterior Descending artery (LAD), the Left Circumflex artery (LCx), and the Right Coronary Artery (RCA). For each vessel the platform reports a standardised FAI score, a unitless value in which raw FAI (the mean attenuation of perivascular adipose tissue, in Hounsfield units, measurements within a radial distance of 3 mm from the outer wall of each vessel) is weighted for technical scan parameters, anatomical factors and basic demographics (age and sex), together with its age-, sex- and vessel-specific centile (described in detail elsewhere [11,17]). In addition to vessel-specific FAI scores, the platform generated an AI-derived CariHeart risk score, which combines the highest FAI score with coronary plaque burden (modified Duke CAD index) and clinical risk factors (diabetes, smoking, hyperlipidaemia and hypertension) to estimate the eight-year risk of cardiac death [7,11].

### 2.5. Risk Stratification Definitions

Conventional CCTA stenosis severity was classified as: no or mild CAD (luminal stenosis < 50%; Coronary Artery Disease Reporting and Data System [CAD-RADS] 0, 1 or 2); moderate CAD (50–69% stenosis, CAD-RADS = 3); or severe CAD (≥70% stenosis including chronic total occlusion, CAD-RADS = 4, 5). For the purposes of the primary analysis, patients were dichotomised into non-obstructive (<50% stenosis) and obstructive (≥50% stenosis) groups. CariHeart risk categories were defined as: low (<1% eight-year cardiovascular mortality); intermediate (1–5%); or high (>5%).

### 2.6. Outcome Measures

The outcome measures were (i) the proportion of patients with non-obstructive CCTA (<50% stenosis) who were classified as high-CariHeart-risk (>5%) and (ii) the change in preventative therapy between CP and NP.

### 2.7. Statistical Analysis

Continuous variables are expressed as mean ± standard deviation (SD) for normally distributed data, or as median with interquartile range (IQR) for skewed distributions, as determined by Shapiro–Wilk testing. Categorical variables are reported as number (n) and percentage (%). Between-group comparisons for continuous variables employed the Mann–Whitney U test; comparisons across three or more CariHeart risk tiers used the Kruskal–Wallis test. Categorical variables were compared using Fisher’s exact test or Pearson’s chi-squared test as appropriate. Recommendations under the CP and NP in the same patients were compared with McNemar’s test for paired proportions. Spearman’s rank correlation coefficient (ρ) was used for bivariate correlation analyses between FAI scores, age, and CAD severity. Associations of the highest FAI score with hypertension, diabetes mellitus and smoking were examined with the Mann–Whitney U test and with linear regression of the log-transformed FAI score adjusted for age and sex. The cross-classification of CCTA grade and CariHeart risk category is presented descriptively. All tests were two-sided, and *p* < 0.05 was considered statistically significant. Statistical analyses were performed using Python 3 (pandas v1.5, scipy v1.10, statsmodels v0.13; Python Software Foundation, USA) and R v4.3 (R Foundation for Statistical Computing, Vienna, Austria); figures were produced using the ggplot2 package.

## 3. Results

### 3.1. Study Population and Baseline Characteristics

Of 137 consecutive patients enrolled, 135 were included in the final analysis; two were excluded due to the absence of contrast-enhanced images. The cohort comprised 63 women (46.7%) and had a mean age of 60.7 ± 11.5 years (range 34–79 years). The majority were of Caucasian ethnicity (n = 111; 82.2%), with smaller proportions identifying as South Asian (n = 9; 6.7%), East Asian (n = 7; 5.2%), Middle Eastern (n = 4; 3.0%), or Black (n = 3; 2.2%); ethnicity was unrecorded in one patient. Atypical chest pain was the predominant presenting symptom in 110 patients (81.5%), with typical angina in 24 (17.8%) and post-COVID-19 breathlessness in one (0.7%).

Cardiovascular risk factors were hypertension in 61 patients (45.2%), dyslipidaemia in 71 (52.6%), family history of premature coronary disease in 73 (54.1%), obesity in 52 (38.5%), diabetes mellitus in 20 (14.8%), and current or former tobacco smoking in 14 (10.4%). Previous ischaemic heart disease had been documented in five patients (3.7%). Baseline characteristics are summarised in Table 1.

### 3.2. CCTA Findings

The majority of patients demonstrated no or mild CAD on CCTA: 112 patients (83.0%) had less than 50% luminal stenosis in all vessels. Moderate CAD (50–69% stenosis) was identified in 21 patients (15.6%), severe CAD was identified (≥70% stenosis) in one patient (0.7%), and one patient (0.7%) had a chronic total occlusion. On a per-vessel basis, obstructive stenosis (≥50%) was identified in 21 LADs (15.6%), 11 of 134 assessable LCxs (8.2%), and 7 of 133 assessable RCAs (5.3%).

### 3.3. FAI Score and CariHeart Risk Score

The median FAI score was 8.6 (IQR 5.0–16.2) for the LAD, 8.6 (IQR 5.2–16.1) for the LCx, and 12.9 (IQR 6.8–27.0) for the RCA. CariHeart risk stratification revealed that only four patients (3.0%) were classified as low-risk (<1% eight-year cardiovascular mortality), 51 (37.8%) as intermediate-risk (1–5%), and 80 (59.3%) as high-risk (>5%). Thus, most patients were classified by CariHeart as having an elevated eight-year risk of cardiac death.

### 3.4. Outcome Measure I. CCTA Grade and CariHeart Risk Category

**Figure 1:**
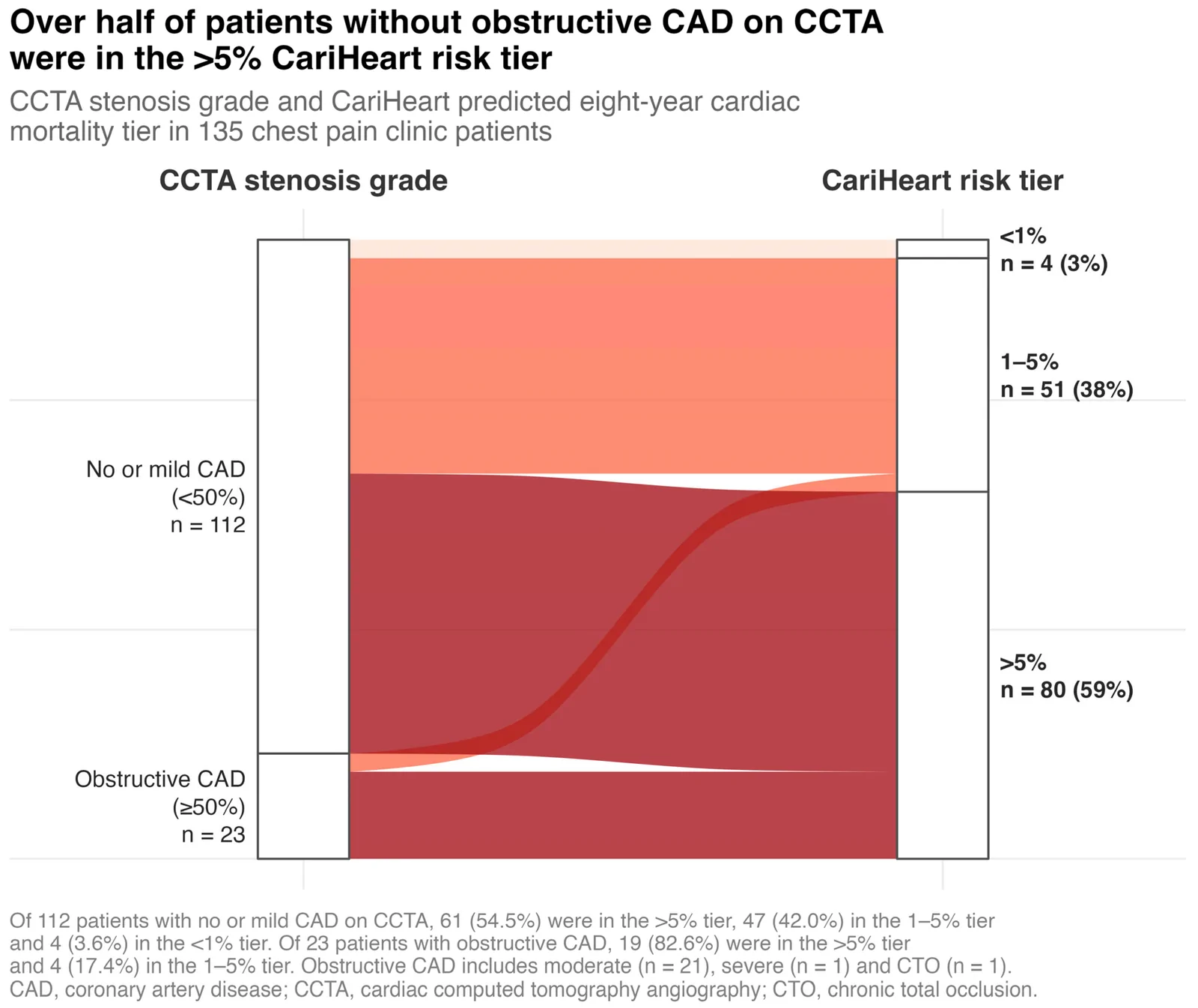
CCTA stenosis grade and CariHeart risk category (eight-year risk of cardiac death) in 135 patients. Ribbon width is proportional to the number of patients. CAD, coronary artery disease; CCTA, Cardiac Computed Tomography Angiography.

Among the 112 patients classified as normal or mild (<50% stenosis) on CCTA, CariHeart risk stratification identified 61 (54.5%) as high-risk, 47 (42.0%) as intermediate-risk, and only four (3.6%) as low-risk (Figure 1). Across the cohort, 61 patients (45.2%) had non-obstructive CAD on CCTA but were classified as high-risk by CariHeart. Conversely, among 23 patients with obstructive CAD on CCTA, 19 were classified as high-CariHeart-risk (82.6%), with the remaining four (17.4%) classified as intermediate-risk.

### 3.5. Clinical Characteristics Associated with High CariHeart Risk Score

Patients classified as having a high CariHeart risk score (n = 80) were significantly older than those at low or intermediate risk (n = 55; mean age 65.9 vs. 53.1 years; *p* < 0.0001) and carried a substantially greater burden of cardiometabolic risk factors. The prevalence of hypertension and diabetes mellitus was higher in the high-risk group (60.0% vs. 23.6%; *p* < 0.0001 and 20.0% vs. 7.3%; *p* = 0.049, respectively). A trend towards higher dyslipidaemia prevalence was also observed (60.0% vs. 41.8%; *p* = 0.053), though this did not reach statistical significance. Obstructive CAD on CCTA was more frequent in the high-CariHeart-risk group (23.8% vs. 7.3%; *p* = 0.018).

By contrast, sex (43.8% vs. 50.9% female; *p* = 0.48), tobacco smoking status (11.2% vs. 9.1%; *p* = 0.78), obesity (38.8% vs. 38.2%; *p* = 1.00), and family history of premature cardiovascular disease (53.8% vs. 54.5%; *p* = 1.00) did not differ significantly between high- and low/intermediate-risk groups. Within the subgroup of patients with non-obstructive CCTA, those classified as high-CariHeart-risk were significantly older (median 66 vs. 53 years; *p* < 0.0001) and had a higher prevalence of hypertension (55.7% vs. 23.5%; *p* = 0.0009) and diabetes mellitus (23.0% vs. 5.9%; *p* = 0.016).

### 3.6. FAI Score Correlates and Inter-Vessel Consistency

**Figure 2:**
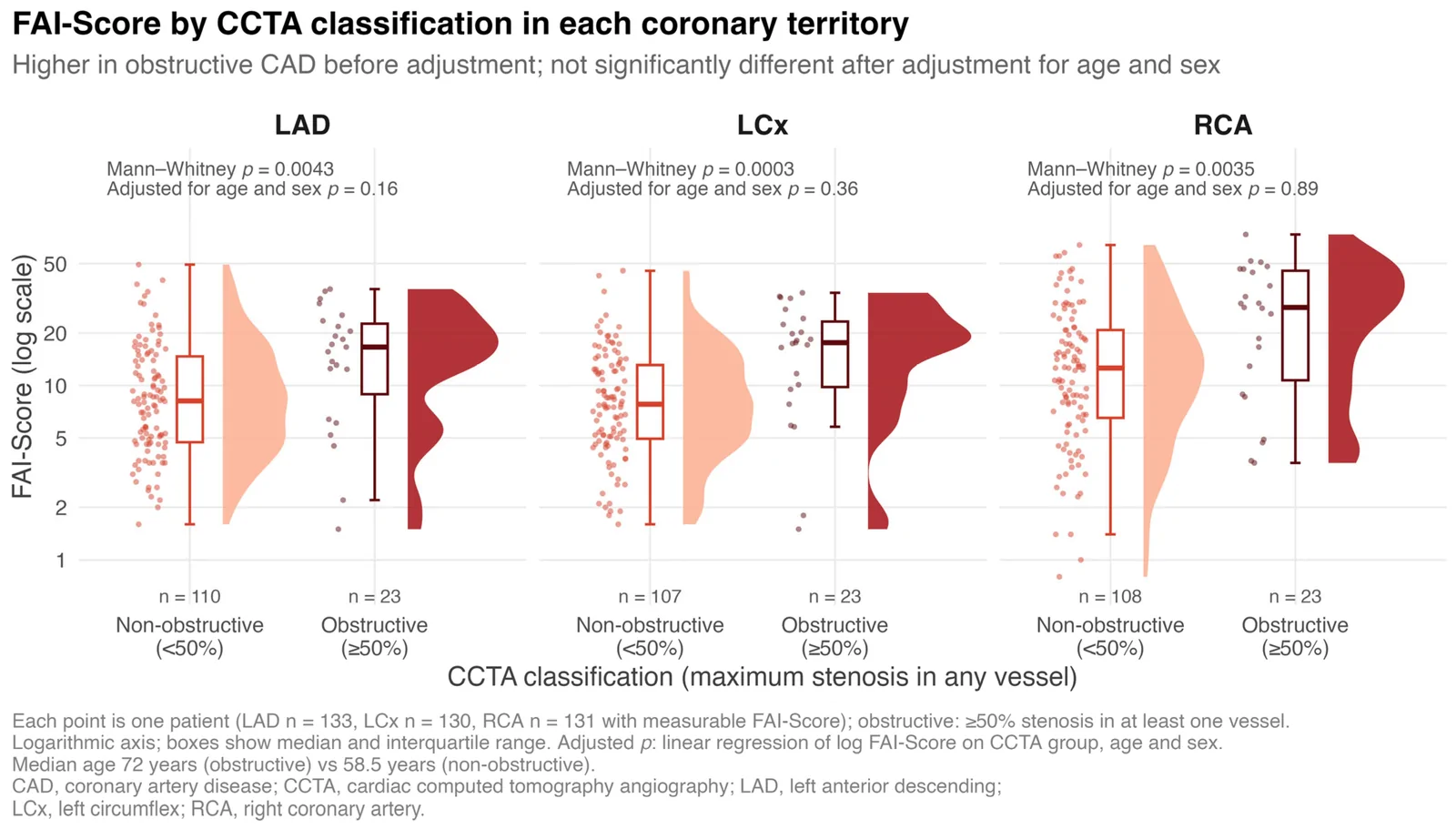
FAI score by CCTA classification in each coronary territory (logarithmic axis); dots represent individual data in a rain cloud plot, boxes show median and interquartile range and the violin density curve on the left of each category. CAD, coronary artery disease; CCTA, Cardiac Computed Tomography Angiography; FAI, fat attenuation index; LAD, Left Anterior Descending artery; LCx, Left Circumflex artery; RCA, Right Coronary Artery.

**Figure 3:**
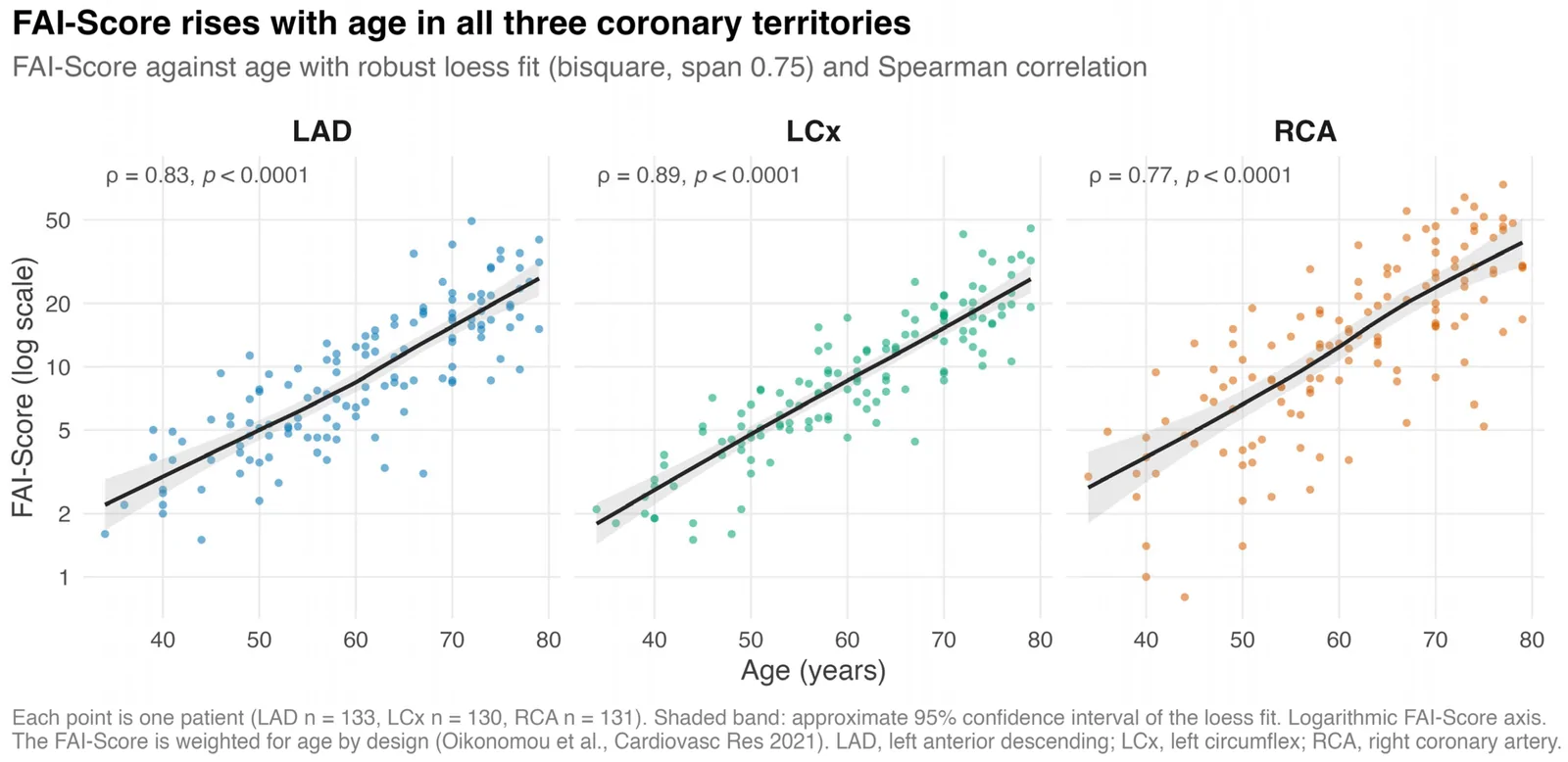
FAI score against age in each coronary territory (logarithmic axis), with robust loess fits and approximate 95% confidence bands. The FAI score is weighted for age by design. FAI, fat attenuation index; LAD, Left Anterior Descending artery; LCx, Left Circumflex artery; RCA, Right Coronary Artery [11].

FAI scores were higher in patients with obstructive compared with non-obstructive coronary anatomy across all three territories: LAD (median 16.6 vs. 8.1; *p* = 0.004), LCx (17.6 vs. 7.8; *p* = 0.0003), and RCA (28.0 vs. 12.6; *p* = 0.004; Figure 2). Strong inter-vessel correlation was observed for all pairings: LAD–LCx (Spearman ρ = 0.913), LAD–RCA (ρ = 0.834), and LCx–RCA (ρ = 0.839; all *p* < 0.0001). Age demonstrated a strong positive correlation with the FAI score in all vessels: LAD (ρ = 0.829), LCx (ρ = 0.891), and RCA (ρ = 0.771; all *p* < 0.0001) (Figure 3). The median FAI score approximately doubled every 11 to 12 years of age, which is similar to the age gradient of the published FAI score nomograms [11]. The highest FAI score did not differ by hypertension (*p* = 0.16), diabetes mellitus (*p* = 0.99) or smoking status (*p* = 0.34), and these comparisons remained non-significant after adjustment for age and sex (*p* = 0.13, 0.92 and 0.97, respectively; Supplementary Table S1).

### 3.7. Outcome Measure II. Change in Preventive Therapy (Current Chest Pain Pathway vs. New Chest Pain Pathway)

**Figure 4:**
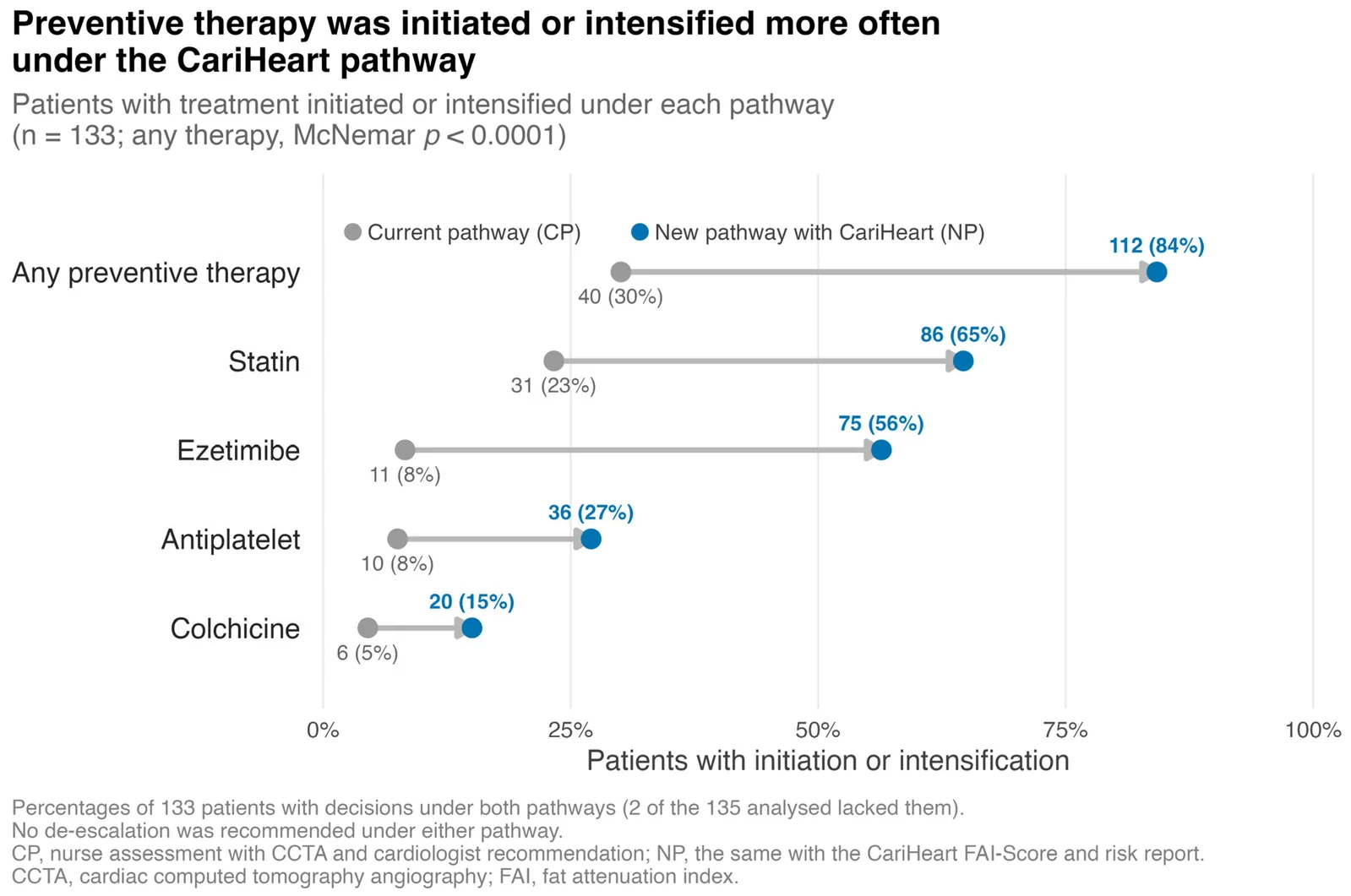
Initiation or intensification of cardiovascular preventive therapy (CVPT) under the current pathway (CP) and new pathway (NP) in 133 patients. CCTA, Cardiac Computed Tomography Angiography; FAI, fat attenuation index.

CariHeart-recommended preventive pharmacotherapy (in agreement with the European Society of Cardiology (ESC) SCORE-based guideline recommendations) for the 133 patients with recommendations included statin therapy in 123 (92.5%), antiplatelet therapy (aspirin) in 108 (81.2%), ezetimibe in 67 (50.4%), and colchicine in 19 (14.3%). These treatment recommendations did not differ significantly between high- and low/intermediate-risk groups, suggesting that the algorithm recommends broad preventive therapy initiation across the cohort. Under the CP, CVPT was initiated or intensified in 40 of 133 patients (30.1%) and under the NP, CVPT was initiated in 112 (84.2%; McNemar *p* < 0.0001); the remaining patients continued their existing therapy, and no de-escalation was recommended under either pathway (Figure 4; Supplementary Table S1). Under the CP and NP, respectively, statins were initiated or intensified in 31 (23.3%) and 86 (64.7%) patients, ezetimibe in 11 (8.3%) and 75 (56.4%), antiplatelet therapy in 10 (7.5%) and 36 (27.1%), and colchicine in six (4.5%) and 20 (15.0%). These counts describe treatments initiated or intensified under each pathway and therefore differ from the treatments listed in the CariHeart report.

## 4. Discussion

### 4.1. Principal Findings

The principal finding of this study is that the newly proposed AI-based pericoronary FAI analysis in addition to the traditional cardiovascular risk factors (captured with the CariHeart risk score) classified a large proportion of patients as high-risk in a contemporary, unselected NHS chest pain clinic cohort. Over half (54.5%) of the patients with anatomically normal or mildly diseased coronary arteries were classified as high-risk (>5% eight-year risk of cardiac death) by CariHeart. This finding is consistent with the CRISP-CT [8] and the ORFAN cohort study [7], which showed that FAI, the FAI score and the AI-risk algorithm that incorporates it predict cardiac mortality and cardiovascular events independently of stenosis, including in patients without obstructive CAD. These patients, under the conventional CP, would have been reassured and discharged without consideration for modification of CVPT.

In ORFAN, making the AI-risk classification available to clinical teams changed management in 45% of 744 consecutive patients in four NHS hospitals [7]. The higher rate of escalation in the present study (84%) may partly reflect differences in definition: we counted initiation or intensification of any of four drug classes, including ezetimibe and aspirin, under a pathway in which the CariHeart report was used directly in the treatment decision.

### 4.2. Implications for Preventive Therapy

However, our study shows that classification as high-risk by CariHeart has direct therapeutic implications for a high proportion of patients. Statin therapy was recommended for 92.5% of patients and colchicine for 14.3%, reflecting the inflammatory risk phenotype identified by the NP. The landmark Canakinumab Anti-Inflammatory Thrombosis Outcome Study (CANTOS) demonstrated that canakinumab, an interleukin-1β antagonist, reduced recurrent cardiovascular events in patients with elevated high-sensitivity C-reactive protein, establishing the principle that targeted anti-inflammatory therapy can reduce coronary event rates [18]. FAI-based risk stratification offers a complementary approach by identifying localised coronary arterial wall inflammation at the point of CCTA analysis, potentially enabling more precise, mechanism-guided preventive intervention, including colchicine, statin intensification, and novel anti-inflammatory agents, in patients who would otherwise be reassured and discharged [7,10,19].

These recommendations are consistent with evidence that the FAI score and CariHeart risk identify patients at increased risk of cardiac events [7,11]. Treatment escalation is, however, a process measure rather than evidence of clinical benefit. Wider use of preventive therapy carries a potential for overtreatment, adverse effects and cost, which were not assessed in this study. Colchicine is not licenced in the United Kingdom for cardiovascular prevention, the role of aspirin in primary prevention is limited, and lipid profiles were not available in this registry to guide the use of ezetimibe; these recommendations therefore require individual clinical judgement and prospective evaluation.

### 4.3. FAI Score, Age and Cardiometabolic Risk Factors

The CariHeart risk score incorporates diabetes, smoking, hyperlipidaemia, hypertension and plaque burden by design [7,11], so the higher prevalence of hypertension and diabetes mellitus among patients classified as high-risk is expected and does not, by itself, indicate a relationship with coronary inflammation. The FAI score, which does not include these risk factors, was not associated with hypertension, diabetes mellitus or smoking in this cohort, either unadjusted or after adjustment for age and sex. These comparisons had limited power, particularly for smoking (14 patients, 10.4%); an association between smoking and perivascular attenuation has been reported in larger datasets [9], and in patients with type 2 diabetes high pericoronary adipose tissue attenuation has been associated with cardiovascular events [20]. The strong correlation between the FAI score and age (ρ = 0.77–0.89) is noted in the developers’ reference population as well and it remains even after adjusting for age [11].

### 4.4. CariHeart Risk and Anatomical CCTA

CCTA stenosis grade and CariHeart risk category measure different constructs, anatomical narrowing and the predicted risk of cardiac death, and were frequently discordant: 61 of 112 patients with non-obstructive CAD were classified as high-risk by CariHeart. This finding is in keeping with the conceptual model that FAI captures a different dimension of coronary risk to that assessed by luminographic stenosis quantification [7,8]. The strong inter-vessel FAI score correlations (ρ = 0.83–0.91) are consistent with a pan-coronary component of perivascular inflammation. This is consistent with the broader body of evidence indicating that inflammation is not simply a consequence of high-grade stenosis but may precede and drive plaque vulnerability and luminal progression [6,8,19].

### 4.5. Ethnic and Demographic Context

The present cohort was predominantly Caucasian (82.2%), with a small but clinically relevant proportion of South Asian (6.7%) and East Asian (5.2%) patients. The CariHeart risk score has been validated predominantly in European-ancestry populations and received regulatory clearance on this basis; its generalisability to non-Caucasian populations requires dedicated evaluation. South Asian individuals are known to have a higher prevalence of both diabetes mellitus and premature coronary artery disease and may carry a disproportionate burden of pericoronary inflammation at younger ages [21]. The limited numbers of non-Caucasian patients in the present study preclude robust ethnic subgroup analysis; this represents an important gap that appropriately powered multiethnic prospective studies should address.

### 4.6. Limitations

Several limitations of this study warrant explicit acknowledgement. First, this is a single-centre registry analysis from one of five NHS sites participating in the NHS England implementation programme of the new AI-based CariHeart risk score, and the findings may not be fully generalisable to all chest pain clinic settings, referral thresholds, or patient populations across the wider population. Second, the study period was limited to nine months (January to September 2024), and the analytic cohort of 135 patients, though consecutive, is modest in size, limiting statistical power for subgroup analyses, particularly in non-Caucasian ethnic groups and tobacco smokers. Third, this is a snapshot cross-sectional analysis; no longitudinal Major Adverse Cardiovascular Event (MACE) outcome data were available during the observation period, precluding any prognostic validation of the CariHeart risk score in this cohort or assessment of whether FAI-based classification translates into improved clinical outcomes in this population. Nevertheless, 10-year outcomes have previously been reported in a large cohort of patients using the ORFAN CCTA database [7]. Consequently, the discrimination and calibration of the CariHeart risk score (for example, ROC-AUC, sensitivity, specificity and calibration) could not be assessed in this cohort. In the development data, predictions moderately overestimated risk at higher levels of predicted risk [11], whereas the external validation in UK patients found the model to be well calibrated [7]; classification above 5% should therefore be read as classification by CariHeart rather than as observed risk. Fourth, whilst the recommended CVPTs were recorded, the present analysis did not assess treatment uptake or physician concordance with recommendations, nor the effect of CVPT on the FAI score. In addition, QRISK3, lipid profiles, baseline statin intensity and coronary calcium scores were not systematically recorded, which limits assessment of whether CP recommendations followed national lipid-modification guidance, and treatment escalation is a process measure rather than evidence of benefit. Finally, no functional imaging data (e.g., CT-derived fractional flow reserve, myocardial perfusion imaging) were available for this cohort; the independent incremental value of FAI over functional assessment could not, therefore, be evaluated.

### 4.7. Future Directions

Adequately powered, prospective, multicentre studies with longitudinal MACE follow-up are required to confirm the prognostic value of CariHeart risk stratification of greater proportions of South Asian and Black patients, groups in whom both the prevalence of cardiometabolic risk factors and the gap in risk stratification tools are greatest [21]. Mechanistic studies examining whether FAI reflects a shared inflammatory substrate across cardiometabolic conditions, including type 2 diabetes, hypertension, and the metabolic syndrome, and whether targeted anti-inflammatory therapy modifies FAI scores and downstream cardiovascular risk would advance both the biological understanding and the therapeutic implications of this imaging biomarker [17,19,22]. The integration of FAI and CariHeart risk score in the current non-invasive anatomical imaging-based chest pain pathway with detailed plaque characterisation, quantitative plaque volume and coronary calcium scoring assessment will provide us with a more accurate risk stratification and disease-modifying medical interventions for our patients referred for assessment of suspected CAD.

## 5. Conclusions

In this single-centre NHS chest pain clinic cohort, CariHeart classified more than half of the patients with non-obstructive CAD on CCTA as high-risk, and the new pathway was associated with a substantially higher rate of recommended initiation or intensification of preventive therapy than the current pathway. These findings support the clinical utility of AI-based CariHeart risk stratification as an adjunct to conventional CCTA in chest pain clinic pathways, enabling identification of a notable residual at-risk population who may benefit from targeted, intensified preventive therapy. The study was retrospective, involved 135 patients at one centre, and did not assess clinical outcomes, treatment uptake or the calibration of the risk estimates; absolute FAI scores also vary with age by design. Future work should report FAI score centiles, record baseline therapy and lipid profiles, follow patients for clinical events and adverse effects of treatment, and evaluate the pathway prospectively across multiple centres and more diverse populations.

## Figures and Tables

**Table 1:** Baseline characteristics of the study cohort (n = 135), overall and by CariHeart risk classification.

Characteristic	Overall (n = 135)	CariHeart High Risk >5% (n = 80)	CariHeart Low/Intermediate ≤5% (n = 55)	*p* Value
**Demographics**				
Age, years, mean ± SD	60.7 ± 11.5	65.9 ± 9.6	53.1 ± 9.7	**<0.0001**
Female sex, n (%)	63 (46.7)	35 (43.8)	28 (50.9)	0.48
Ethnicity, n (%)				
Caucasian	111 (82.2)	69 (86.2)	42 (76.4)	0.17
South Asian	9 (6.7)	5 (6.2)	4 (7.3)	1.00
East Asian	7 (5.2)	4 (5.0)	3 (5.5)	1.00
Black	3 (2.2)	0 (0.0)	3 (5.5)	0.065
Middle Eastern	4 (3.0)	2 (2.5)	2 (3.6)	1.00
Unknown	1 (0.7)	0 (0.0)	1 (1.8)	0.41
**Presenting symptom**				
Atypical chest pain, n (%)	110 (81.5)	63 (78.8)	47 (85.5)	0.37
Typical angina, n (%)	24 (17.8)	16 (20.0)	8 (14.5)	0.50
Post-COVID-19 breathlessness, n (%)	1 (0.7)	1 (1.2)	0 (0.0)	1.00
**Cardiovascular risk factors, n (%)**				
Hypertension	61 (45.2)	48 (60.0)	13 (23.6)	**<0.0001**
Dyslipidaemia	71 (52.6)	48 (60.0)	23 (41.8)	0.053
Family history of premature CVD	73 (54.1)	43 (53.8)	30 (54.5)	1.00
Obesity (BMI > 30 kg/m^2^)	52 (38.5)	31 (38.8)	21 (38.2)	1.00
Diabetes mellitus	20 (14.8)	16 (20.0)	4 (7.3)	**0.049**
Smoking (current or ex)	14 (10.4)	9 (11.2)	5 (9.1)	0.78
Previous ischaemic heart disease	5 (3.7)	4 (5.0)	1 (1.8)	0.65
**CCTA findings, n (%)**				
No or mild CAD (<50%)	112 (83.0)	61 (76.2)	51 (92.7)	**0.018**
Moderate CAD (50–69%)	21 (15.6)	17 (21.2)	4 (7.3)	**0.031**
Severe CAD (≥70%)	1 (0.7)	1 (1.2)	0 (0.0)	1.00
Chronic total occlusion	1 (0.7)	1 (1.2)	0 (0.0)	1.00
Any obstructive CAD (≥50%)	23 (17.0)	19 (23.8)	4 (7.3)	**0.018**
**Pericoronary FAI score, median (IQR)**				
LAD	8.6 (5.0–16.2)	13.9 (8.2–20.3)	5.1 (3.6–8.0)	**<0.0001**
LCx	8.6 (5.2–16.1)	13.5 (7.8–18.1)	5.1 (3.5–7.8)	**<0.0001**
RCA	12.9 (6.8–27.0)	21.5 (12.9–33.6)	6.8 (3.9–11.2)	**<0.0001**
**CariHeart risk classification, n (%)**				
Low risk (<1%)	4 (3.0)	–	–	–
Intermediate risk (1–5%)	51 (37.8)	–	–	–
High risk (>5%)	80 (59.3)	–	–	–

Values are n (%), mean ± standard deviation, or median (interquartile range). Percentages use the analysed cohort (n = 135) as the denominator; two of 137 enrolled patients were excluded for absent intravenous contrast. *p* values compare high risk (>5%) with low/intermediate risk (≤5%): Mann–Whitney U test for continuous variables and Fisher’s exact test for categorical variables. Bold *p* values are significant at *p* < 0.05. FAI score is available in 133 (LAD), 130 (LCx) and 131 (RCA) patients. BMI, Body Mass Index; CAD, coronary artery disease; CCTA, Cardiac Computed Tomography Angiography; CVD, cardiovascular disease; FAI, fat attenuation index; IQR, interquartile range; LAD, Left Anterior Descending artery; LCx, Left Circumflex artery; RCA, Right Coronary Artery; SD, standard deviation.

## Data Availability

The data presented in this study are available on reasonable request from the corresponding author. The data are not publicly available due to patient confidentiality requirements.

## Supplementary Materials

The following supporting information can be downloaded at: https://www.mdpi.com/article/10.3390/jcm15218639/s1, Table S1: Patient characteristics by escalation of cardiovascular preventive therapy under the current chest pain pathway (CP) and the new CariHeart-risk score based pathway (NP).
