# Supplementary material for: Computed Tomography Angiography-Derived Pericoronary Fat Attenuation Index and AI-Based Cardiovascular Risk Score as a Proposed New Pathway in a Rapid Access Chest Pain Clinic: A Single-Centre Retrospective Registry Study
Source: J Clin Med. 2026 Nov 6;15(21):8639. doi: 10.3390/jcm15218639 (PMC13764554; doi:10.3390/jcm15218639)
Supplement: Supplementary file 1 [file jcm-15-08639-s001.zip › jcm-4636067-supplementary.pdf]

Supplementary Table S1. Patient characteristics by escalation of cardiovascular preventive therapy under the current chest pain pathway (CP) and the new CariHeart-risk score based pathway (NP); n = 133.

	Current pathway (CP)			New pathway with CariHeart (NP)		
Characteristic	No change (n = 93)	Started or intensified (n = 40)	p	No change (n = 21)	Started or intensified (n = 112)	p
Patients, n (%)	93 (70)	40 (30)		21 (16)	112 (84)	
**Demographics**						
Age, years, median (IQR)	58.0 (50.0–70.0)	64.5 (57.0–73.5)	**0.024**	70.0 (52.3–74.0)	61.0 (51.0–70.0)	0.16
Male sex, n (%)	45 (47.9)	26 (65.0)	0.070	8 (38.1)	63 (56.2)	0.13
Body surface area, m², median (IQR)	1.90 (1.72–2.01)	1.99 (1.80–2.05)	0.27	1.83 (1.63–2.00)	1.90 (1.79–2.05)	**0.044**
Body mass index, kg/m², median (IQR)	27.8 (24.4–32.6)	28.1 (26.0–31.3)	0.95	26.9 (25.2–29.5)	28.6 (25.4–32.8)	0.16
**Cardiovascular risk factors, n (%)**						
Hypertension	40 (42.6)	21 (52.5)	0.29	11 (52.4)	49 (43.7)	0.47
Dyslipidaemia	49 (52.1)	23 (57.5)	0.57	15 (71.4)	56 (50.0)	0.072
Diabetes mellitus	15 (16.0)	5 (12.5)	0.61	5 (23.8)	15 (13.4)	0.22
Smoking (current or ex)	11 (11.7)	3 (7.5)	0.47	1 (4.8)	13 (11.6)	0.35
Obesity (BMI >30 kg/m²)	38 (40.4)	14 (35.0)	0.56	6 (28.6)	46 (41.1)	0.28
Family history of CVD	50 (53.2)	22 (55.0)	0.85	11 (52.4)	61 (54.5)	0.86
**Baseline medications, n (%)**						
ACE inhibitor / ARB	25 (26.6)	15 (37.5)	0.21	8 (38.1)	32 (28.6)	0.38
Aspirin / clopidogrel	58 (61.7)	31 (77.5)	0.078	18 (85.7)	71 (63.4)	**0.046**
Calcium-channel blocker	27 (28.7)	15 (37.5)	0.32	8 (38.1)	33 (29.5)	0.43
Nitrate	16 (17.0)	9 (22.5)	0.46	2 (9.5)	23 (20.5)	0.24
Beta-blocker	42 (44.7)	15 (37.5)	0.44	9 (42.9)	48 (42.9)	1.00
**RACPC assessment, n (%)**						
Typical angina	16 (17.0)	8 (20.0)	0.68	5 (23.8)	19 (17.0)	0.45
**CCTA result (local report), n (%)**						
No or mild CAD (<50%)	84 (89.4)	28 (70.0)	**0.006**	16 (76.2)	95 (84.8)	0.33
Moderate CAD (50–69%)	9 (9.6)	11 (27.5)	**0.008**	5 (23.8)	15 (13.4)	0.22
Severe CAD (≥70%)	1 (1.1)	1 (2.5)	0.53	0 (0.0)	2 (1.8)	0.54
**Change in preventive therapy, n (% of all 133 patients)**						
Statin started	0 (0.0)	31 (23.3)		0 (0.0)	36 (27.1)	
Statin intensified	0 (0.0)	0 (0.0)		0 (0.0)	50 (37.6)	
Statin started or intensified	0 (0.0)	31 (23.3)		0 (0.0)	86 (64.7)	
Statin maintained	57 (42.9)	7 (5.3)		18 (13.5)	26 (19.5)	
Statin started, intensified or maintained	57 (42.9)	38 (28.6)		18 (13.5)	112 (84.2)	
Ezetimibe started	0 (0.0)	11 (8.3)		0 (0.0)	75 (56.4)	
Colchicine started	0 (0.0)	6 (4.5)		0 (0.0)	20 (15.0)	
Antiplatelet started	0 (0.0)	10 (7.5)		0 (0.0)	36 (27.1)	
**Pericoronary FAI-Score, median (IQR)**						
LAD	7.7 (4.6–14.4)	13.0 (8.1–20.0)	**0.001**	8.4 (4.9–15.9)	9.1 (5.1–16.0)	0.65
LCx	7.8 (4.8–13.5)	12.0 (7.5–19.7)	**0.002**	9.1 (4.7–16.2)	8.6 (5.2–16.3)	0.82
RCA	10.5 (5.4–18.4)	21.2 (11.8–36.1)	**<0.001**	15.6 (6.7–24.5)	12.9 (7.0–26.6)	1.00
**Vessel with highest FAI-Score, n (%)**						

Characteristic	Current pathway (CP)			New pathway with CariHeart (NP)		
	No change (n = 93)	Started or intensified (n = 40)	p	No change (n = 21)	Started or intensified (n = 112)	p
LAD	18 (19.2)	4 (10.0)	0.19	2 (9.5)	20 (17.9)	0.35
LCx	17 (18.1)	5 (12.5)	0.42	6 (28.6)	16 (14.3)	0.11
RCA	58 (61.7)	31 (77.5)	0.076	13 (61.9)	76 (67.9)	0.59
**FAI percentile in highest-FAI vessel**						
FAI percentile, median (IQR)	92.0 (77.5–97.0)	95.5 (88.5–99.0)	**0.010**	82.0 (70.8–95.0)	93.0 (84.5–98.0)	**0.006**
Low tier: ≤50th centile (n = 4), n (row %)	4 (100)	0 (0)	0.18	2 (50)	2 (50)	0.057
Intermediate tier: 50–75th LAD/RCA or 50–90th LCx (n = 23), n (row %)	20 (87)	3 (13)	0.050	5 (22)	18 (78)	0.39
High tier: >75th LAD/RCA or >90th LCx (n = 106), n (row %)	69 (65)	37 (35)	**0.016**	14 (13)	92 (87)	0.11
**CariHeart risk score tier, n (row %)**						
Low risk, <1% (n = 4)	3 (75)	1 (25)	0.82	1 (25)	3 (75)	0.61
Intermediate risk, 1–5% (n = 50)	42 (84)	8 (16)	**0.006**	10 (20)	40 (80)	0.30
High risk, >5% (n = 79)	48 (61)	31 (39)	**0.005**	10 (13)	69 (87)	0.23
**CariHeart-reported CAD grade, n (row %)**						
No or mild CAD (<50%) (n = 114)	86 (75)	28 (25)	**0.002**	15 (13)	99 (87)	0.060
Moderate CAD (50–69%) (n = 13)	5 (38)	8 (62)	**0.007**	4 (31)	9 (69)	0.11
Severe CAD (≥70%) (n = 3)	1 (33)	2 (67)	0.15	1 (33)	2 (67)	0.38
None reported (n = 3)	1 (33)	2 (67)	0.35	1 (33)	2 (67)	0.38

Values are n (%) or median (interquartile range). n = 133 patients with complete pathway recommendation data (of 135 analysed). "Started or intensified" denotes any cardiovascular preventive therapy initiated or intensified under that pathway; "No change" denotes none. Percentage conventions: unmarked sections use column percentages; "Change in preventive therapy" uses percentages of all 133 patients; sections marked n (row %) use row percentages within each category. p values compare the no-change and started/intensified groups within each pathway; the therapy-change rows, which define the groups, carry no p values (Mann-Whitney U test for continuous variables; Fisher's exact or chi-squared test for categorical variables). Red bold p values are significant at $p < 0.05$. No therapy de-escalation was recommended in either pathway. ACE, Angiotensin-Converting Enzyme; ARB, Angiotensin Receptor Blocker; BMI, Body Mass Index; CAD, Coronary Artery Disease; CCTA, Cardiac Computed Tomography Angiography; CVD, Cardiovascular Disease; FAI, Fat Attenuation Index; IQR, Interquartile Range; LAD, Left Anterior Descending artery; LCx, Left Circumflex artery; RACPC, Rapid-Access Chest Pain Clinic; RCA, Right Coronary Artery.
